# Discussions and perspectives regarding oxytocin as a biomarker in human investigations

**DOI:** 10.1016/j.heliyon.2021.e08289

**Published:** 2021-10-30

**Authors:** Juan Carlos Caicedo Mera, Melissa Andrea Cárdenas Molano, Christian Camilo García López, Cristina Acevedo Triana, Jorge Martínez Cotrina

**Affiliations:** Laboratorio Interdisciplinar de Ciencias y Procesos Humanos LINCIPH, Facultad de Ciencias Sociales y Humanas, Universidad Externado de Colombia, Colombia

**Keywords:** Oxytocin, Saliva, ELISA, RIA, Social behavior

## Abstract

This article introduces a review of research that has implemented oxytocin measurements in different fluids such as plasma, cerebrospinal fluid, urine and, mainly, saliva. The main purpose is to evaluate the level of evidence supporting the measurement of this biomarker implicated in a variety of psychological and social processes. First, a review of the technical developments that allowed the characterization, function establishing, and central and peripheral levels of this hormone is proposed. Then, the article approaches the current discussions regarding the level of reliability of the laboratory techniques that enable the measurement of oxytocin, focusing mainly on the determination of its concentration in saliva through Enzyme-Linked Immunosorbent Assay (ELISA). Finally, research results, which have established the major physiological correlates of this hormone in fields such as social neuroscience and neuropsychology, are collected and discussed in terms of the hormone measurement methods that different authors have used. In this way, the article is expected to contribute to the panorama of debates and current perspectives regarding investigation involving this important biomarker.

## 1 Introduction

In recent years, the measurement of different biomarkers has taken a main role in research on physiological correlates of different phenomena such as stress, aggressiveness, prosocial and affiliative behaviors, moral judgement and development, social emotions, among others. One of the molecules whose study has generated high interest is oxytocin (OXT). This nonapeptide is one of two neuro-hormones released by the posterior hypophysis, along with arginine vasopressin (AVP). OXT is produced by the supraoptic and paraventricular nuclei of the hypothalamus and then transported to the gland by structures denominated tuber cinereum and infundibulum (Gounden et al., 2021). It has a main role in the feminine reproductive system, since it engages in mechanisms modulating the activity of the uterine smooth muscle during processes such as childbirth (Takagi et al., 1985; Takeda et al., 1985; Kuwabara et al., 1987) and myometrial contraction during menstruation, in addition to the activation of milk producing lactiferous sinuses in mammary glands (Carter et al., 2007; Grewen et al., 2010; Magon and Kalra, 2011; Martino, 2014). In clinical settings, it constitutes a fundamental tool for labor induction, given its capacity to increase the frequency, duration and intensity of contractions once labor has spontaneously started (OMS, 2015). It is also fundamental to achieve the increase in uterine muscular tone immediately after birth, reducing the risk of bleeding associated with hypotonia (Gimpl and Fahrenholz, 2001).

Nowadays, OXT levels determination are used beyond the investigations involving reproductive physiology. Probably linked with these functions that are crucial for survival in mammals, OXT is also involved in several social and affective processes. OXT has been widely studied due to its role as a facilitator of maternal care behaviors in many mammal species and has also been associated with trait attitudes of paternal behavior, that, depending on the species, have great relevance in offspring care (Barg, 2011). It has been established that receptors of oxytocin (OXT) and arginine vasopressin (AVP) neuropeptides are distributed in different brain regions associated with central nervous system control of stress, anxiety and social behavior (Landgraf and Neumann, 2004). In humans, OXT correlates with the expressions of reciprocity in interaction, social recognition and social bonding (Magon and Kalra, 2011). In a review, Zik and Roberts (2015) further associated it with the establishment of affective links and affiliative behaviors (trust, generosity, cooperation, social hugs and emotional empathy), but also with social exclusion and envy, revealing that the behavioral role of this hormone could be more complex and context dependent than it seemed before.

Over the past decades, saliva has been the fluid in which many efforts to measure OXT have focused, in order to establish non-invasive correlates of these psychological and physiological functions. Nevertheless, measurements of physiological levels of this hormone have been difficult, due to its small molecular size and homology with other peptides.

In such a context, the purpose of this narrative review is to focus on the different techniques that allow the measurement of OXT and current discussions of its validity, mainly in the field of social neuroscience and affective neuroscience. In order to avoid possible biases associated to the search of results in scientific literature, this review included papers that reported positive and negative results related to the validity in OXT measurements. The search criteria for articles were broad, based on key words such as Oxytocin measurements, saliva samples, plasma, urine, spinal fluid, ELISA, EIA and RIA. In some cases, relevant original articles referenced in different papers or reviews were searched and revised, mainly those that involved protocols and measurement techniques of OXT. Databases used in this review were PubMed, Scopus, ScienceDirect, SciELO and Google Scholar.

## 2 Development of oxytocin detection techniques

There are different approaches to detect and quantify OXT. Since its discovery in 1909 by Henry Dale, the peptides detection was performed in pituitary tissue, which was carefully selected to collect only the posterior part of the gland. Then, the macerate was processed in order to remove any blood residue and finally, through Biuret's reagent, the peptides presence in the sample were evidenced (Dale, 1909).

The investigation on the peptide's functions required its extraction and, at the time, it was not yet identified as a biomarker. However, in 1928 the peptide was separated from vasopressin at Parke-Davis & Co Research Laboratory (Rowe, 1928). Further investigations showed how oxytocin was extracted and inoculated in animal models exvivo, in order to analyze its function. Hence, evidence started to build in oxytocin's activity and its differences with vasopressin (David and Vareed, 1929). It was not until 1928 that investigation with the peptide was applied to humans. Burne and Burn tested the action of OXT extracted from the pituitary gland in the human uterus during childbirth (David and Vareed, 1929). In 1940, Rosenfeld extracted the molecule by centrifugation. Applying a gravitational force of 250000g for 6 h, after boiling the sediment in diluted acetic acid, two types of sediments appeared. Later, in 1952, Schiebler observed the sediments through electronic microscopy. Thus, the sediments were found to be composed of granules from 50nm to 200nm, corresponding to OXT and AVP (Barer et al., 1963).

In addition to the extraction of the nonapeptide via acetic acid, other techniques such as gel filtration and ether-acetone (Bisset and Walker, 1954 cited by Chard et al., 1970) were used. On the other side, electrophysiological recordings started to reveal what the peptide caused compared to the action of different drugs (Daniel and Wolowyk, 1966), evidencing the existence of oxytocin receptors at a peripheral level, which were later studied through techniques such as autoradiography (Sato et al., 1984).

The field of study of peptides released by the pituitary gland had its turn in the seventies, when an interest to quantify them started to emerge. Barer et al. (1963), quantified OXT and AVP in rabbits making comparisons between the amount of substance in adult males, nursing females and offspring. Barer and his colleagues applied the combined technique of homogenization, centrifugation and hormonal granules or sediment detection by electronic microscopy. They noticed that nursing females had an increase in OXT (1691mU) in comparison with the OXT found in adult males.

A fusion between extraction and immunoassay techniques, allowed a better precision in the analysis and quantification of OXT (Chard et al., 1970). The Radioimmunoassay (RIA), was initially developed by Salomon Berson & Rosalyn Yalow, in 1960, in order to detect concentrations of a certain substance in a sample in the magnitude of picograms. It is a very demanding procedure; therefore, its use is limited to certain investigations where a low number of samples must be processed and in small quantities (Chard et al., 1970). Additionally, the life span of the reagent is short and the equipment required is considerably expensive, as well as the implements and regulatory biosecurity controls for the manipulation of the radioactive isotope (Voller et al., 1978).

In contrast, the sensitivity of the Enzymatic Immuno Assay technique (EIA), developed by Van Weemen and Schuurs in 1971, was not as thorough as RIA; however, it did not have as many disadvantages as the second. In parallel, during that same year the Swedish scientists Engyvall and Perlmann developed the Enzyme-Linked ImmunoSorbent Assay or ELISA. These investigators, driven by the search of an alternative to RIA, created a technique that had better outcomes in contrast with other immunoassays, it even opened the door for a possible substitution of RIA, as Ekins and Watson explained in “ELISA: a replacement for radioimmonoassays?” (1976). Despite the similarities between both techniques, ELISA changed the radioactive isotope that harmed the laboratories budget and eliminated the risk for the researchers working with these reagents (Szeto et al., 2011). Other benefits were associated with the ease of use of the reagents that, at the time, were planned to be distributed in a commercial kit (Ekins and Watson, 1976).

The antibody specification for both techniques was improved through the years and it did not take long before it was used for the detection and quantification of oxytocin. Chard et al. (1970) were pioneers in the implementation of RIA for the detection and quantification of the peptide in human plasma. After proving the sensitivity of the assay (1,5 pg/ml), they noticed that in males, not pregnant women and women in the first trimester of pregnancy did not show more than 1,5 pg/ml in blood serum. Burgeon et al. (1991) evaluated the O13 with RIA, ELISA and immunohistochemistry techniques. On one hand, the results showed that O13 was very specific for OXT through the ELISA technique. On the other hand, researchers noticed that not all monoclonal antibodies bonded with the OXT tagged with the radioactive isotope. These findings would help solve the discussion about the effectiveness of ELISA compared with RIA, especially in samples with low OXT. The findings mentioned above supported, directly or indirectly, the development of salivary OXT measurement. However, further research, cited by Robinson et al. (2014), which demanded noninvasive methods for the assessment of systematic levels of the nonapeptide, directed the search towards less harmful alternatives.

## 3 Discussions around validity of oxytocin measurements in different fluids

Kramer et al. (2004) published a paper describing an enzyme immunoassay method for quantifying plasma OXT in two different species: Sprague Dawley rats and prairie voles, using a commercial EIA kit. They specified that the antibody recognized the oxidized active form of OXT. The assay had an OXT minimum detection limit of 4,68 pg/ml and had cross-reactivity with similar neuropeptides found in mammalian sera at less than 0.001%. A series of plasma dilutions resulted in a displacement curve parallel to the standard curve of the kit and accuracy tests resulted in a high correlation between expected and observed values. In addition, injection with OXT resulted in a significant increase of plasma OXT. They concluded that the EIA is valid and can be used to reliably measure plasma OXT concentrations, describing some of the advantages this method has over available RIA kits. For instance, authors reported studies demonstrating that EIA tended to yield higher values of OXT than RIA, and it could be associated to the fact that RIA may also have more restrictive detection limits for OXT.

In 2007, authors of the same research group were pioneers in making an effective measurement of salivary oxytocin using ELISA, modifying the sample in order to concentrate it and facilitating the subsequent analysis. The main advantage of this type of measurement is the non-invasive procedure for taking the sample, which reduces ethical impediments in non-clinical research and contributes to reduce the possible noise of venipuncture stress. They reported a higher antibody specificity for OXT while RIA showed more cross-reactivity with AVP. The authors’ findings support that OXT is present in human saliva, showing a consistent variation pattern of salivary OXT with lactation (with ranges of nearly 4 pg/ml during the feeding and nearly 10 pg/ml 30 min prior to feeding). They also tested changes in salivary OXT after a massage in males, reporting a range of variation between 1,75 before and 2,1 pg/ml after the massage, which follow the variation pattern measured in plasma OXT: 175 pg/ml before and 220 pg/ml after the massage, nearly one order of magnitude above (Carter et al., 2007).

However, these results contrasted what was discussed in 2005 by Horvat-Gordon, Granger, Schwartz Nelson and Kivlighan, who denied the soundness of the measurement of salivary OXT and its correlational inaccuracy with behavior. Nonetheless, Carter and colleagues approached the problem modifying the sample, drying it through vacuum centrifugation, and then hydrating it in order to make it four times more concentrated. Experimentally, they performed a comparison between recovered OXT in saliva vs. blood, before and after a massage treatment in males. The results showed consistency in the increase of OXT taken after the treatment in both blood (pre – 175 pg/ml and post – 225 pg/ml) and saliva (pre – 1,3 pg/ml and post – 2 pg/ml) (Carter et al., 2007). Following research, continued the protocol developed by Carter (Hoffman et al., 2012; Holt-Lunstad et al., 2011), where a correlation between changes in salivary OXT and behavior was shown.

Other attempts to measure salivary OXT were executed through the ELISA technique in addition to the treatment of vacuum centrifugation implemented by Carter, but the results were still paradoxical, as was evidenced in “Correlation of plasma and salivary oxytocin in healthy young men – Experimental evidence” by Javor et al. (2014). In contrast, there is evidence that points towards the effectiveness of salivary oxytocin measurement (MacLean et al., 2017, 2018; White-Traut et al., 2009). Facing these inconsistencies, Horvat-Gordon et al. (2005) proposed four arguments why OXT might not be a good biomarker since it is a salivary measurement. (1) The weight of OXT is higher than most hormonal biomarkers measured in saliva (e.g. cortisol, testosterone) and in combination with its short lifespan, it might mean there is not an adequate transport from blood to saliva. (2) The concentrations of other biomarkers in saliva tend to be 5–10% compared to its measurement in blood. (3) The enzymes present in saliva could help degrade OXT faster before its measurement. (4) The OXT levels detected could get to be false positives due to the contamination of saliva by blood or other compounds (Behr et al., 2017). On the contrary MacLean et al. (2019) points out four considerations for the measurement of OXT in saliva and other peripheral fluids that need to be explored in further investigations: 1) The correlation with central measurements 2) temporal resolution and the biological clearance 3) biding with other components and 4) the presence of interferences during measurement.

As was implied, being able to assess the relation between central OXT from peripheral measurements (e.g. blood, saliva) is crucial to understand the effect of this hormone in social behavior or to postulate its usefulness as a clinical marker in different psychological or psychiatric disorders (McQuaid et al., 2014; Woolley et al., 2014; Lebowitz et al., 2016). The effort to relate levels of central OXT with peripheral OXT is mainly driven by the simplification of the method, since sample collection is considerably simpler and less invasive peripherally (preferably through saliva), in comparison with sample collection at a central level (lumbar puncture in order to extract cerebrospinal fluid; CSF). Yamamoto et al. (2019) show that peripheral OXT is transported through the blood-brain barrier into the brain by the receptor for advanced glycation end-products (RAGE). This evidence supports the idea that central and peripherical concentrations of OXT are connected and that one may affect the other.

The evidence on evaluation of peripheral oxytocin as an indicator of concentrations at a central level is contradictory. There are diverse physiological stimuli that relate to the increase of oxytocin production at both central and peripheral levels, for instance, during pregnancy or childbirth, during nursing and during sexual intercourse. In several of these conditions, an increase in the oxytocin concentration in CSF and plasma has been found, although they are not necessarily correlated (Neumann and Landgraf, 2012).

Likewise, there are studies performed in humans in which a positive relation is found between oxytocin levels in CSF and plasma (Wang et al., 2013; Carson et al., 2015). In a review by Valstad et al. (2017) it was found that, in general, in human and other species studies there is a positive correlation between the values of oxytocin in CSF and plasma when there is an intranasal administration of oxytocin (IAO) or a stress-inducing situation, although there is not a relation in basal levels.

In contrast, there are studies in humans in which a relation was not found between the central and peripheral values (Takagi et al., 1985; Takeda et al., 1985; Jokinen et al., 2012; Kagerbauer et al., 2013; Striepens et al., 2013; Martin et al., 2014) (view Table 1). These negative results may be due to the role of the blood-brain barrier, because it limits the free transit of cerebral OXT to the blood tract or vice versa (Neumann and Landgraf, 2012). So, it is possible to conclude that there is still no definitive consensus of this topic.

**Table 1:** Comparison between different studies with measurements of the oxytocin (OXT) levels in different fluids (CSF, plasma, saliva and urine).

Author	n	Sex	Age	Detection technique	Fluid measured	Statistical analysis	Statistical report
Takagi et al. (1985)	36	F	19–45	RIA	CSF/Plasma	Correlation	NS
Takeda et al. (1985)	42	M F	±30 - ± 40	RIA	CSF/Plasma	Correlation	NS
Holt-Lunstad et al. (2008)	72	M F	20–39	EIA	Plasma/Saliva	Correlation	*p* < 0.01
Feldman et al. (2010)	112	M F	±30	ELISA	Plasma/Saliva	Spearman Correlation	*p* < 0.001
Grewen et al. (2010)	20	M		EIA	Plasma/Saliva	Pearson Correlation	*Basal p* = 0.022
Feldman et al. (2011)	112	M F	±30	ELISA	Plasma/Saliva/Urine	Pearson Correlation	*p*OXT & *s*OXT (p < 0.001) *p*OXT & *u*OXT (NS) *s*OXT & *u*OXT (NS)
Hoffman et al. (2012)	20	M	±30	EIA	Plasma/Saliva/Urine	Pearson Correlation	*p*OXT & *s*OXT (NS) *p*OXT & *u*OXT (NS)
Jokinen et al. (2012)	47	M F	23–66	RIA	CSF/Plasma	Spearman Correlation	NS
Kagerbauer et al. (2013)	41	M F	19–81	RIA	CSF/Plasma	Spearman Correlation	NS
Striepens et al. (2013)	15	M	19–64	RIA	CSF/Plasma	Spearman Correlation	NS
Wang et al. (2013)	215	M F	20–62	RIA	CSF/Plasma	Linear regression	*p* < 0.001
Carson et al. (2015)	27	M F	4–64	EIA	CSF/Plasma	Linear regression	*p* = 0.0064
Javor et al. (2014)	30	M	18–40	ELISA	Plasma/Saliva	Correlation/linear regression	NS
Martin et al. (2014)	41	M F	19–58	RIA	CSF/Plasma	Spearman Correlation	NS
Behr et al. (2017)	38	M	18–45	ELISA	Serum/Saliva	Spearman Correlation	NS
Martin et al. (2018)	50	M F	20–86 (±65)	RIA	Plasma/Saliva/CSF	Spearman Correlation	*s*OXT & *c*OXT (*p* < 0.001) *p*OXT & *c*OXT (*p = 0.003*) *s*OXT & *p*OXT (*p* = 0.10)

*Notes*: ***F*** female, ***M*** male, ***CSF*** cerebrospinal fluid, ***NS*** non-significant, ***pOXT*** plasmatic oxytocin, ***sOXT*** salivary oxytocin, ***uOXT*** urine oxytocin, ***cOXT*** central oxytocin.

Regarding the diverse peripheral measurements and the link between them, there is currently no definitive consensus either. There are studies in which no relation between salivary and plasmatic OXT is found (Javor et al., 2014) or between salivary and blood serum (Behr et al., 2017). Furthermore, Robinson et al. (2014) found that investigators must be cautions when correlating the results of OXT in saliva and blood obtained through ELISA, since there are variations depending on the sample collection technique. Other authors have also reported difficulties in validating OXT measurements with different techniques and in different types of biological fluids (McCullough et al., 2013).

Likewise, urine use has been attempted as a peripheral measurement of OXT levels, although more evidence is required to implement it as a reliable measurement. Hoffman et al. (2012) have found, excluding some outliners, a moderate positive correlation between OXT levels in plasma and urine. On the other hand, Feldman et al. (2011) found no correlation between OXT levels in urine in comparison with the ones found in plasma or saliva.

In contrast, other studies suggest that it is possible to presume hormonal measurements results as valid in different fluids and to establish correlations between them. For instance, Martin et al. (2018) found that peripherical measures of OXT had a positive correlation with OXT in CSF. The correlation between salivary and central OXT was stronger (rho = 0.657; *p* < 0.001) than plasma and CSF (rho = 0.417; p = 0.003), and even more than that between plasma and salivary OXT (rho = 0.361; p = 0.010). In studies performed in humans, a positive relation between plasmatic and salivary oxytocin levels was found (Holt-Lunstad et al., 2008; Feldman et al., 2011; Grewen et al., 2010). A study in patients with nervous anorexia showed that plasmatic and salivary OXT were positively correlated (Hoffman et al., 2012).

## 4 Functional correlates of oxytocin in the social and psychological sphere

Different reviews have pointed that OXT appears to enable animals to overcome the natural proximity avoidance in order to inhibit defensive behavior, which facilitates approaches to others (Uvnäs-Moberg, 1998). This is accomplished due to the fact that OXT shows a significant association with the limbic system, including the amygdala, associating it with a decrease in anxiety and the neuroendocrine response to stress in social interactions (Neumann and Landgraf, 2012). In general, while the central activity that mobilizes AVP appears to be associated with an increase in surveillance, anxiety, excitation and activation, OXT has behavioral and neural effects related to anxiety reduction, relaxing, growth and restauration (Carter et al., 2007). Thus, it has been found that OXT reduces endocrine effects and psychological responses to social stress, modulates social memory and increases trust, generosity and the capacity to infer others’ mental states (Heinrichs and Domes, 2008). Studies also have demonstrated that OXT is associated with different dimensions of social relationships, including parental attention and care, involvement with peers, sexual behavior, and the development of memory and social cognition (Uvnäs-Moberg, 1998). That is why, in humans, OXT was known initially as the “love and union hormone”, involving in different types of affective processes such as romantic bond, social bond, compassion and empathetic behaviors (Magon and Kalra, 2011).

Considering controversial findings regarding validity of OXT measurements set out above, social and behavioral correlates of OXT levels reported in different investigations will be discussed (See Table 2).

**Table 2:** Summary of methods, main findings and ranges of variation reported in different studies dealing with social and psychological correlates of OXT.

Research topic	Author	Fluid measured	Detection technique	Validated protocol	Main findings	Approximate range of variation of OXT (Units may differ)
OXT and maternal/parental behavior	Levine et al. (2007)	Plasma	EIA	Kramer et al. (2004)	Increase in OXT throughout pregnancy. This ascending pattern is associated with a greater maternal-fetal bond.	45–550 pg/ml
	Feldman et al. (2007)	Plasma	EIA	Kramer et al. (2004)	OXT levels at the postpartum strongly predicted maternal behavior in association with other variables such as attachment representations and maternal preoccupation.	50–550 pM
	Nishizato et al. (2017)	Saliva	ELISA	Carter et al. (2007)	OXTs have a prominent effect on social stimulus attention in younger infants.	70–350 pg/ml
	Rybicka et al. (2021)	Saliva	RIA	Neumann et al. (2013)	Women's OXT level rises in response to a doll's cry.	NR
	Moussa et al. (2021)	Saliva	ELISA	Carter et al. (2007)	Increase in salivary OXT in mothers and their children with a healthy bond, when the mothers gave them a massage.	10–360 pg/ml
	Gordon et al. (2010)	Plasma	EIA	Kramer et al. (2004)	OXT predicts proximity, touch, and gaze behavior in mother-father-infant ecological interactions.	200–500 pg/ml
	Gettler et al. (2021)	Saliva	ELISA	Carter et al. (2007)	Fathers who had both an increase in salivary OXT and a decrease in testosterone at the time of holding the infant on the first day of birth, showed postpartum bonding behaviors.	70–100 pg/ml
	Nawa et al. (2020)	Saliva	ELISA	Feldman et al. (2014)	Maternal psychological distress and trauma related to the experience of a natural disaster were negatively associated with increased levels of OXT, after a playful interaction.	8–12 pg/mg salivary protein
	Light et al. (2000)	Plasma	RIA	Amico et al. (1985)	The mothers' contact with their babies, after having been subjected to a stress-inducing protocol, generated an increase in OXT and a decrease in blood pressure.	NR
	Lebowitz et al. (2016)	Saliva	ELISA	Carter et al. (2007)	There was a negative correlation between levels of familiar adjustment to support the youth with anxiety symptoms and the reduction of OXT levels in this population.	0–70 pg/ml
OXT and romantic attachment	de Jong et al. (2015)	Saliva	RIA	de Jong et al. (2015)	OXT levels are increased significantly with respect to basal measurements after running, sexual self-stimulation and the stress induced task, but not in response to breastfeeding.	0–30 pg/ml
	Weisman et al. (2013)	Plasma	ELISA	Carter et al. (2007)	Men showed significantly higher mean OXT than women. Higher OXT in women correlated with greater attachment anxiety.	100–800 pg/ml
	Marazziti et al. (2006)	Plasma	RIA	Marazziti et al. (2006)	Attachment anxiety and OXT are positively associated in romantic bond.	0,13–4,59 pg/ml
	Schneiderman et al. (2012)	Plasma	ELISA	Carter et al. (2007)	OXT was significantly higher in new lovers compared to singles and correlated with the couples' interactive reciprocity.	250–509 pg/ml
	Ditzen et al. (2007)	Plasma	RIA	Landgraf et al. (1995)	Women with positive physical partner contact before stress induction, exhibited significantly lower cortisol and heart rate responses to stress but plasma OXT levels did not change.	NR
	Gonzaga et al. (2006)	Plasma	RIA	Demitrack et al. (1990); Weitzman, Glatz, and Fisher, (1978)	In the context of an auto-biographic emotional evocation task that included affiliation cues and sexual cues, only the former correlated positively with OXT.	NR
OXT and stress response	Opacka-Juffry & Mohiyeddini. (2012)	Plasma	ELISA	As described by the manufacturer	High levels of stressful situations during adolescence are related with lower levels of OXT in plasma during adulthood. OXT had a negative relationship with the presence of psychological disorders as depression and anxiety.	78,6–1198 pg/ml
	Thomas & Larkin. (2020)	Plasma	ELISA	NR	Patients diagnosed with major depressive disorder had lower levels of OXT compared with a control group. There was no correlation between oxytocin and cortisol.	20–420 pg/ml
	Kuchenbecker et al. (2021)	Saliva	ELISA	As described by the manufacturer	Increased academic stress was associated with higher levels of OXT in female college students.	0–35 pg/ml
	McQuaid et al. (2016)	Saliva	ELISA	Carter et al. (2007)	There was a negative correlation between salivary OXT and cortisol in plasma, in women who were submitted to a social stress protocol.	5–13 pg/ml
	Bernhard et al. (2018)	Saliva	RIA	de Jong et al., (2015)	Participants who underwent a social stress protocol had a moderate correlation between salivary OXT and cortisol, as well as negative correlations between OXT and anxiety and insecurity.	1,3–2,7 pg/ml
	Glenk et al. (2020)	Plasma	ELISA	Carter et al. (2007)	Allergy sufferers had higher basal levels of OXT than non-allergy sufferers. After undergoing a social stress protocol there were no significant intra- or intergroup statistical changes.	700–1400 pg/ml
	Chiodera et al. (1991)	Plasma	RIA	NR	Increase of OXT and a synchronic reduction of ACTH and cortisol, which had a similar pattern but appeared earlier in lactating women compared to no-lactating women who received breast stimulation.	2–15 pmol/l
OXT and psychopathology	Turner et al. (1999)	Plasma	RIA	Demitrack et al. (1990), Weitzman et al. (1978)	Women who showed increased OXT levels for positive emotions and massages, and who maintained OXT levels during negative emotions reported less interpersonal problems associated with intrusiveness.	4-5,6 pg/ml
	Levy et al. (2015)	Saliva	ELISA	Carter et al. (2007)	An inverse correlation was found between salivary OXT levels and Callous unemotional behaviors	0,36–14,14 pg/ml
	Fujisawa et al. (2014)	Saliva	ELISA	Carter et al. (2007)	Levels of oxytocin in saliva are positively associated to the gaze fixation time of images containing people interacting and showing emotions in children with normal development. In contrast, autistic children do not show the same correlation.	15–70 pg/ml
	Lebowitz et al. (2019).	Saliva	ELISA	Carter et al. (2007)	Higher levels of salivary OXT were associated with negative social bonds and in turn with suicidal ideation	4,5–75,8 pg/ml
	Rubin et al. (2013)	Plasma	EIA	Carter et al. (2007)	Peripheral vasopressin but not OXT relates to severity of acute psychosis in women with acutely-ill untreated first-episode psychosis.	100–1000 pg/ml
Others stimulus associated to OXT secretion	Procyshyn et al. (2020)	Saliva	ELISA	Carter et al. (2007)	After exposing to an empathy inducing video about the story of a gravely ill child, high OXT responders had greater levels of empathy.	80–160 pg/ml
	Bellosta-Batalla et al. (2020)	Saliva	ELISA	Carter et al. (2007)	A brief mindfulness session induced an increase in salivary OXT.	140–230 pg/ml
	Rassovsky et al. (2019)	Saliva	ELISA	As described by the manufacturer	There was a significant increase in salivary OXT immediately after a high-intensity Jujitsu training. Effect was stronger in response to close tactile contact interaction during the practice.	27–58 pg/ml
	Tarumi and Shinohara (2020)	Saliva	ELISA	Carter et al. (2007)	There was a significant increase of OXT after smelling different essential oils such as lavender, neroli, jasmine absolute, among others.	NR
	Geva et al. (2020)	Saliva	ELISA	Carter et al. (2007)	Contact with a robot that was designed to elicit a feeling of social connection, was associated to mood improvements, but reduced salivary OXT levels in adults.	22–30 pg/ml

Note: NR (Not reported).

## 5 OXT and maternal/parental behavior

As already stated, the first findings about OXT functions dealt with reproductive physiology in mammals. However, recent studies in humans reveal new insights in this field. In an investigation with 66 pregnant women, Levine et al. (2007), measured OXT in blood samples at three different moments: Time-I: early pregnancy (at the end of first trimester); Time-II: early third trimester pregnancy; and Time-III: within the first month postpartum. The authors reported five typologies of OXT variation along the three times, with the lowest levels of OXT at Time-I (45 pg/ml) and the highest levels at Time-III (550 pg/ml). They also applied the Maternal-Fetal Attachment Scale (MFAS) and founded that the increase in OXT from early to late pregnancy was correlated with a higher maternal–fetal bonding. Plasma levels of OXT were determined using the mentioned EIA protocol validated by Kramer et al. (2004) in rodent models.

The same research team developed another investigation in 2007 where they measured plasma oxytocin and cortisol levels of 62 pregnant women during the same three periods: first trimester, last trimester, and first postpartum month. After the infants were born, they observed interactions between mothers and their children, and the mothers were interviewed about their infant related thoughts and behaviors. Results showed that, whilst repeated measures analysis of variance revealed that cortisol levels changed significantly across the study period, OXT levels had a stable trend across time. On the other hand, oxytocin levels at early pregnancy and the postpartum period were associated to maternal bonding behaviors such as gaze, vocalizations, positive affect and frequent checking of the infant, as well as attachment-related thoughts. In a multiple regression model, OXT levels at the postpartum strongly predicted maternal behavior in association with other variables such as attachment representations and maternal preoccupation. Authors used again Kramer et al. (2004) protocol. In this work they reported results in picomolar concentration and obtained a range of variation similar to the one showed by the same authors in the paper cited above (between 50 and 550 pM.), in which they used pg/ml units (Feldman et al., 2007).

To study the possible role of OXT in social cues attention in early life stages, Nishizato et al. (2017) examined visual attention using an eye tracking system in infants and children (5–90 months of age) and measured the concentration of OXT in saliva samples. They reported a negative association between age and both attention toward social cues and salivary OXT levels. They also found a positive association between age and attention for non-social cues. These were able to reflect a differential role of OXT in attention mechanisms depending on the age of children, with a salient effect over social stimulus attention only in the younger ones. Authors used the salivary OXT ELISA method developed by Carter et al. (2007) reporting levels of OXT of about 70 pg/ml, with a higher level in younger children that reached nearly 350 pg/ml.

On another investigation, Rybicka et al. (2021) indicated that OXT modulates parental behaviors related to breastfeeding, including parental sensitivity to infant crying. Authors exposed women to a crying doll and they showed an increase in their salivary OXT levels. This effect was also found when they are in a couple. These oxytocin levels were measured in saliva according to Neumann et al. (2013), by means of a radioimmunoassay where the detection limit was 0.1–0.5 pg/sample (Rybicka et al., 2021).

Some behaviors related to mother-infant bonding may also be mediated by OXT. Moussa et al. (2021), reported an increase in salivary OXT in mothers who did a massage session to their infants, but this increase only happened when this bond was healthy (p = 0.05). In contrast, mothers without a healthy bond with the infant did not show such an increase (p = 0.398). Additionally, an increase in salivary OXT levels was reported in children whose attachments were healthy following massage provided by the mother (p = 0.041) (Moussa et al., 2021). Saliva samples were processed as cited in Carter et al. (2007), by immunoassay and the minimum detection limit was reported to be 4.68 pg/ml (Carter et al., 2007; Moussa et al., 2021).

In order to the test possible role of OXT as a mediator in the triadic interactions between mother, father and infant, Gordon et al. (2010), measured plasma OXT in 37 couples and their firstborn infant through a two times home-visit protocol. At six months, they videotaped and micro-coded patterns of proximity, touch, and gaze behavior in mother-father-infant ecological interactions whilst they were playing together. Authors found that the synchrony in these behavioral measurements defined as moments of coordination between physical proximity and affectionate touch was predicted by both maternal and paternal OXT. Again, these authors used the EIA Kramer et al. protocol, reporting mean variations between 298,51 and 327,33 pg/ml between the first and second visit.

Father-infant bonding may also be modulated by OXT. Gettler et al. (2021) reported that fathers who had both an increase in salivary OXT and a decrease in testosterone at the time of holding the infant on the first day of birth, showed postpartum bonding behaviors such as play to a greater extent than fathers who had elevated levels of both hormones. In addition, OXT levels before holding the infant for the first time were found to be 73.5 pg/ml and then upon holding the infant increased to 89.12 pg/ml. The researchers showed that the physical contact between parent and infant leads to a significant increase in salivary OXT (∗p < .001). Saliva samples were processed by immunoassay kit according to the protocol of Carter et al. (2007). A sensitivity of 15 pg/ml was reported and the coefficient of variation was <21.7% interassay and <15% intraassay (Gettler et al., 2021).

These results highlighted the importance of OXT in the modeling of the attachment bond and maternal/parental care behavior. However, less is known about possible adverse factors that could change or attenuate these hormonal effects. In a recent study (Nawa et al., 2020), 34 mother-child dyads who suffered the Great East Japan Earthquake in 2012, received in 2015 a protocol of playful interaction, measuring OXT levels before and after intervention. The children's average age at the moment of study was 8,8 years old. A significant increase in maternal OXT levels was detected following playful interaction, and this effect was more intense among mothers of first-born boys. However, maternal psychological distress and trauma related to experiencing a natural disaster were negatively associated with an increase of OXT levels. Authors measured OXT through an ELISA kit less used than the one mentioned above, following the protocol of Feldman et al. (2014). In addition, although this method allows the measure of OXT levels in units of pg/ml, in this study the data was normalized by protein concentration in the saliva (pg/mg salivary protein), obtaining a narrower range of variation between 8 to 12 pg/mg protein (Nawa et al., 2020).

Light et al. (2000), evaluated blood OXT changes in lactating mothers after a stress protocol based on a speech task. Half of them could hold their babies finishing the stress induction, and the other half had no contact with their babies. Authors reported three patterns of hormonal responses: OXT decrease, minimal OXT change and OXT increase. The group which reached an increase higher than 100% OXT change from baseline was the mothers with increased pattern and baby contact condition. Women with increased pattern but no baby contact condition only reached a small percentage increase of change from baseline (less than 20 %). In addition, the mother's group with higher change levels displayed blood pressure reduction, reflecting a possible anxiolytic effect of peripheric OXT elevation. Authors used an RIA protocol developed by Amico et al. (1985) with a minimal detectable oxytocin concentration in extracted plasma of 0.5 pg/ml. However, results with this method are expressed in micro-Units per milliliter (uU/ml), which is not common in recent studies. In fact, Light et al. do not report absolute values of OXT but only percent of change in OXT from baseline for each hormone responsivity group. It becomes more difficult to evaluate these results in terms of data validity and to compare them with other investigations.

Finally, some authors have shown that OXT is not only involved in early bonding between children and caregivers, but also in anxiety due to separation of emotional ties in young people. However, it is sensitive to the adjustments of families facing this kind of anxiety as well. This, since a negative correlation was found between levels of familiar adjustment to support the youth with anxiety symptoms and the reduction of OXT levels in this population (Lebowitz et al., 2016). In this research, authors used the salivary ELISA method validated by Carter et al. (2007) and the range of variation in OXT levels reached up to 70 pg/ml. These values seem low compared to ranges in lactating women or people who are starting a romantic relation, and even to ranges of men and women from a general population sample reported by Weisman et al. (2013) whose mean was 375.78 pg/ml.

According to the findings above, behaviors that underlie bonding in families are related to an increase of OXT and this hormone could be playing a role as a biomarker to detect troubles in the parents' and children's interactions.

## 6 OXT and romantic attachment

OXT has been also involved in romantic bonding, showing its importance in different affective and affiliative processes. On one hand, de Jong et al. (2015) measured salivary OXT in healthy adult males and females who performed different activities: physical exercise (running), sexual self-stimulation including orgasm, breast-feeding, and a stress inducing protocol: Trier Social Stress Test (TSST). They demonstrated that OXT levels are increased significantly with respect to basal measurements after running, sexual self-stimulation and the stress induced task, but not in response to breastfeeding. These authors also used a RIA protocol to measure OXT with a detection limit in the range of 0,5 pg, and intra- and inter-assay variabilities of <10%. However, data ranged from 0 to 10 pg/ml in the sexual self-stimulation and running conditions, 0–15 pg/ml in TSST and 0–30 pg/ml in the breastfeeding condition. In other words, sharing the same units, variation order is 10 times lower compared to reports of plasma measurements. This order of variation of salivary OXT is consistent with that reported by Carter et al. (2007) in their article on the validation of an ELISA method.

On the other hand, studying a big sample of subjects (N = 473), Weisman et al., 2013a, Weisman et al., 2013b measured plasma OXT in order to describe the distributions of plasma OXT in women and men, and to examine whether the relations between OXT and two types of anxiety (trait and attachment anxiety) are moderated by gender. Results indicated a mean of 375.78 pg/ml, SD = 264.03 with a distribution trend bias toward high values. Men showed significantly higher mean OXT than women (women: 327.13 pg/ml, SD = 164.43; men: 399.91, SD = 183.65; t = 2.57, p = .01). Regarding the possible association with anxiety, trait anxiety was lower only among men with higher OXT. In contrast, higher OXT in women correlated with greater attachment anxiety. Authors used the aforementioned ELISA protocol validated by Carter et al. (2007).

In another study exploring romantic attachment anxiety, forty-five healthy subjects filled the Italian version of the "Experiences in Close Relationships" (ECR), a self-report questionnaire to evaluate romantic attachment. Using the OXT RIA kit with a sensitivity of 10–30 pg/tube, authors found that attachment anxiety and OXT are positively associated in romantic bond to a statistically significant degree (r = 0.30, p = 0.04). Range of variation of OXT level in this study appears unusually low: 0,13–4,59 pg/ml (Marazziti et al., 2006).

To assess the role of OXT in romantic attachment, Schneiderman et al. (2012) examined plasma OXT in 120 new lovers (60 couples) three months after the initiation of their romantic relationship and 43 non-attached singles. Authors followed the 25 couples who stayed together six months later. Dyadic interactions were systematically observed and interviews regarding relationship-related thoughts and behaviors were made. OXT was significantly higher in new lovers compared to singles, p < .001, which could show its role in maintaining romantic bond at least in the initial stages. These high levels of OXT among new lovers did not decrease six months later and showed high individual stability. In addition, OXT correlated with the couples’ interactive reciprocity, including positive affect, affectionate contact and empathetic worry about anxiety of the partner. OXT levels at the beginning of the relation also allow to differentiate couples who remained together from those who did not. In this research authors used the already mentioned ELISA protocol developed by Carter et al. (2007) to measure OXT, reporting a range of variation between 250 pg/ml (in single subjects) and 509 pg/ml (in lovers). These values of OXT elevation are similar to those reported in parents of newborns.

Conversely, in a study carried out by Ditzen et al. (2007) 67 women aged 20–37 years old were exposed to a standardized psychosocial laboratory stressor (Trier Social Stress Test). Prior to the stress induction, 25 of women had no partner interaction, 22 had verbal social support, and 20 received physical contact (standardized neck and shoulder massage). Women with positive physical partner contact before stress exhibited significantly lower cortisol and heart rate responses to stress but plasma OXT levels did not change. Authors used a RIA method to measure OXT with a minimal detection limit in the range of 0.1 pg/sample (Landgraf et al., 1995). However, they do not report absolute values of OXT and only describe absence of significant statistical differences between groups.

Gonzaga et al. (2006) analyzed, in the context of an auto-biographic emotional evocation task, reports of 26 women in terms of affiliation cues and sexual cues, and searched for correlations with changes in plasma OXT levels. They found that only affiliation cues correlated positively with OXT. The authors mentioned that they used a RIA technique with a sensitivity of 2,1 pg/ul; however, they reported results in terms of correlation orders and not in absolute values, which makes the comparison of hormone values with other investigations more difficult. Taken together, these results show that OXT is related to different psychological expressions of a romantic bond, such as, attachment anxiety, reward, social support and sexual arousal.

## 7 OXT and stress response

It has been found that both neurohormones, OXT and vasopressin (AVP), interact with the hypothalamic-pituitary-adrenal axis (HPA) and the stress response (McQuaid et al., 2014). While the AVP correlates with an increase of the adrenocortical hormone (ACTH) (Legros, 2001) and the activation of the HPA, OXT is associated with an inhibition of the HPA and the secretion of ACTH (Legros, 2001) and cortisol (McQuaid et al., 2016).

Opacka-Juffry and Mohiyeddini (2012) found that high levels of stressful situations during adolescence are related with lower levels of OXT in plasma (M = 377,6 ± 23,9 pg/ml [78.6–1198 pg/ml]) during adulthood. They have also found a negative relationship between OXT and the presence of affective psychological disorders as depression and anxiety. Authors used an ELISA kit with a sensitivity of 11,7 pg/ml.

In the same way, Thomas and Larkin (2020) found no correlation between oxytocin and cortisol, nevertheless, they found that patients diagnosed with major depressive disorder had lower levels of OXT (M = 158 pg/ml ± 140) compared with a control group (M = 261.7 pg/ml ± 158.97). A negative correlation was also found between OXT levels and depression, and the stress scale in the depression anxiety and stress scales (DASS). To measure OXT, authors mentioned they used an ELISA kit with a sensitivity of 15 pg/ml, but they did not specify the manufacturer or validated protocol.

Furthermore, Kuchenbecker et al. (2021) compared the changes in cortisol and OXT, in saliva, across an academic semester in women. They found that mid-semester (M = 7.58 ± 3.52) the OXT levels were lower than late-semester (M = 22.66 ± 9.15). This showed that the increased academic stress was related with higher OXT levels (F (1,28) = 36.44; *p* < 0,001). Authors used Carter et al. (2007) ELISA protocol.

To assess the effect of acute stress on OXT secretion, McQuaid et al. (2016) made a study in which women were submitted to the Trier Social Stress Test (TSST). They found a negative correlation (r (55) = -0.28; *p* = 0.03) between salivary OXT, measured through an ELISA kit following the protocol of Carter et al. (2007), and cortisol in plasma using a radioimmune assay (RIA). There were no differences between the control group (M = 10.54 ± 4.98), and the stress group with social support (M = 10.97 ± 1.11), and the stress group without social support (M = 12.06 ± 0.96) in OXT levels. It is striking that the range of variation of OXT in this study was between 5 and 13 pg/ml, which is relatively low compared to other studies that used the same protocol to measure salivary OXT.

Bernhard et al. (2018) found that participants who submitted to the same protocol of stress (TSST) presented a moderated correlation between salivary OXT and cortisol (+1 min: r = 0.31, p = .10; +10 min: r = 0.37, p = .05; +25 min: r = 0.38, p = .05; +55 min: r = 0.35, p = .07), and negative correlations between OXT and anxiety (*r* = -0.40, *p* = 0.04), and insecurity (*r* = -0.36, *p* = 0.04). They used a RIA protocol developed by Jong et al. (2015) with a detection limit in the 0.1–0.5pg/sample range. The OXT values in this study appear relatively low, moving in a range of 1,3–2,7 pg/ml.

Although most studies point to an inverse correlation between OXT and stress, and cortisol, there evidence is mixed. For instance, Glenk et al. (2020) found that allergic people had higher basal levels of OXT (M = 1187.62 ± 256.59 pg/ml) than nonallergic people (M = 997.74 ± 230.65; *t* (34) = 2.335, *p* = 0.077, *d* = -0.651). However, after being submitted to the TSST they found no significant statistical changes intra or intergroups. Authors used an ELISA kit based in Carter et al. (2007) protocol, with a detection limit of 15 pg/ml.

Finally, to explore the relation between a physiological condition that induces OXT and a possible stress reduction, one of the first studies in this topic evaluated changes in OXT, Cortisol and ACTH after suckling (in lactating women) and breast stimulation (in no lactating women). Authors measured these hormones through a RIA protocol with a detection limit of 2 pmol/l for OXT. Results showed an increase of OXT and a synchronic reduction of ACTH and cortisol, which had a similar pattern but appeared earlier in lactating women compared to no-lactating women who received breast stimulation. Authors reported their results in units pmol/l, with a range of variation of OXT between 2 and 15 (Chiodera et al., 1991). In comparison with plasma OXT levels reported in pg/ml, this range of variation is two order of magnitude lower.

## 8 OXT and psychopathology

Different researches relate peripheral oxytocin with different psychiatric disorders such as psychopathy, anxiety, depression, autism and others (Rutigliano et al., 2016), or some of its clinical manifestations.

In order to evaluate the effect of OXT in mood changes, Turner et al. (1999) studied twenty-five normal cycling healthy women who performed an imagination task and completed questionnaires on attachment and interpersonal problems, followed by three interventions: massage, positive emotion evocation, and negative emotion evocation. Results showed a tendency for OXT to be elevated in response to relaxation massages and decreased in response to sad emotions. Women who showed increased OXT levels for positive emotions and massages, and who maintained OXT levels during negative emotions reported less interpersonal problems associated with intrusiveness. On the other hand, women who were in a relationship had greater increases in OXT in response to positive emotions. However, higher basal levels of OXT were associated with greater interpersonal distress. In this paper, authors used a RIA method with a sensitivity of 2.1 pg/ml. They reported results in pg/ml, but despite carrying out blood measurements of OXT, values ranged from 4 to 5,6 pg/ml. These values and the magnitude of the change are very low compared to other studies.

Personality profiles with lack of empathy and emotional insensitivity (Callous-unemotional) are related to antisocial behaviors (Mann et al., 2015). Levy et al. (2015), sought correlations between salivary OXT and Callous-unemotional behaviors in adolescents diagnosed with antisocial behavior problems. An inverse correlation was found between salivary OXT levels and Callous unemotional behaviors (p ≤ 0.05). This research group used an immunoassay according to (Carter et al., 2007) (sensitivity of 15 pg/ml). These results show that the use of oxytocin levels found in saliva as a biomarker can be possible to detect Callous unemotional traits and antisocial behaviors (Levy et al., 2015).

On the other hand, levels of oxytocin in saliva are positively associated to the gaze fixation time of images containing people interacting and showing emotions in children with normal development. In contrast, autistic children do not show the same correlation (Fujisawa et al., 2014). It was reported that children with normal development had 44.5 pg/ml of oxytocin in saliva and children diagnosed within the autistic spectrum had 39.33 pg/ml of the neuropeptide. This analysis was performed by immunoassay according to Carter et al. (2007).

Additionally, there is a correspondence between anxiety, suicidal ideation and negative social interactions, and this correlated with salivary OXT levels (Lebowitz et al., 2019). Lebowitz et al. (2019), used an immunoassay to measure salivary OXT according to protocols established by Carter et al. (2007) (sensitivity of 15 pg/ml). They found that higher levels of salivary OXT were associated with negative social bonds and in turn with suicidal ideation (CI: 0.81 to 2.25, p = <0.001).

Despite the above findings, there is still a wide controversy discussing whether this peptide may be used as a biomarker for detection of psychiatric diseases. Some meta-analyses point out that it is not reliable, regardless of the type of sample taken (Rutigliano et al., 2016). For example, a study carried out by Rubin et al. (2013) showed that Peripheral vasopressin but not OXT relates to severity of acute psychosis in women with acutely-ill untreated first-episode psychosis. Authors measured plasma levels hormones using a EIA kit as validated by Carter et al. (2007). They describe that EIA is highly sensitive (minimal detection levels:12 pg/ml OXT; 4 pg/ml AVP) and specific with cross-reactivity between OT and AVP of 0.04%.

## 9 Others stimulus associated to OXT secretion

Recent studies have remarked other possible stimulus able to induce OXT secretion. Procyshyn et al. (2020) measured salivary OXT and testosterone in 173 healthy adults before and after showing an empathy inducing video about the story of a gravely ill child. Results showed a group of subjects high OXT responders and other group high testosterone responders, with greater levels of empathy in the first one. Again, the authors used Carter et al. (2007) ELISA protocol. The values of OXT they found ranged from 80 to 160 pg/ml.

On the other hand, Bellosta-Batalla et al. (2020), evaluated the impact of a brief mindfulness session on mood and salivary OXT in psychology students. They found an increase in OXT levels in group received intervention, as well as a reduction in negative affect and anxiety. With the afore mentioned Carter et al. (2007) ELISA protocol and they reported the highest values of about 230 pg/ml in the experimental group.

In a research with 68 beginner and advanced practitioners of Jujitsu, a form of traditional martial arts originating in Japan, Rassovsky et al. (2019) found a significant increase in salivary OXT immediately after a high-intensity training, returning to baseline levels following a cool-down period. Additionally, they reported a significantly higher increase in salivary OXT in response to close tactile contact interaction during the practice. They found a range of variation between 27 – 58 pg/ml, using a commercial OXT Enzyme-Linked Immunosorbent Assay (ELISA) kit. Authors did not report the minimal detection level of the kit they used, but mentioned that the intra-assay coefficients of samples and controls were less than 12.4% and 14.5%, respectively.

In another study, Tarumi and Shinohara (2020) tested possible effects of different essential oils on salivary OXT concentration in postmenopausal women. They found a significant increase of OXT after smelling lavender, neroli, jasmine absolute, roman chamomile, clary sage and Indian sandalwood, and no changes in OXT levels in the control group exposed to dipropylene glycol. Again, they used Carter et al. (2007) ELISA protocol but did not report absolute values. The oxytocin concentration was expressed as “rate of change”, which makes the comparison of results with other studies harder.

Finally, in a recent article Geva et al. (2020) tested a protocol of human-robot social touch, using a robot named PARO, which has characteristics of tenderness, a kind and soft surface and has a structure similar to that of a baby seal. It was designed to elicit a feeling of social connection. Paradoxically, contact with this robot was associated to mood improvements, but reduced salivary OXT levels in adults. This research was carried out using Carter et al. (2007) protocol, and the range of variations was between 22 – 30 pg/ml.

## 10 Studies using intranasal administration of OXT

Over the past decades, many researchers rather evaluate the effect of exogenous administration of OXT on behavior and mental states, instead of measuring peripheric hormone concentration or induce secretion through physiological stimulus such as the mentioned above. Intranasal administration of OXT (IAO) is the most common way to deliver the hormone, with a low-risk procedure based of a nasal spray. It has been shown that neuropeptides go through the blood-brain barrier after intranasal administration, which provides a useful method to study OXT effects on the central nervous system in humans (Heinrichs and Domes, 2008).

IAO has been related to an increase in plasmatic and central levels in humans (Striepens et al., 2013; Wang et al., 2013), and in rats and mice (Neumann et al., 2013). However, Freeman et al. (2016), report that IAO is only meaningful at a plasmatic level when it is over 1IU/kg in Rhesus monkeys, but there are no significant changes in CSF. In contrast, when the administration is intravenous, there is an increase related with the administered dose as an effect in CSF and plasma. Lee et al. (2018) showed that after intranasal and intravenous administration of OXT in rhesus macaques CSF there was an increase of artificial OXT in CSF, but there was no release of endogenous OXT.

On the other hand, an increase in salivary oxytocin has been found after IAO (Burri et al., 2008; Gossen et al., 2012; van Ijzendoorn et al., 2012; Weisman et al., 2012a, Weisman et al., 2012b; Daughters et al., 2015; Russell et al., 2018). Although there is strong evidence on the link between IAO and salivary oxytocin levels, these results have to be interpreted carefully since this increase in salivary concentrations might be present due to a step of the artificial drug from the nasal cavity into the oropharynx (Quintana et al., 2018; Fragkaki et al., 2020). This is reflected on the high values of salivary OXT in comparison with baselines or control groups (McCullough et al., 2013). Recently, some authors have proposed a set of strategies to improve the precision of IAO investigations, by the complementary consideration of methodology (in terms of verifying that intranasal administration increases central levels of oxytocin), theory (generating robust models of the role of oxytocin in behavior and physiology) and reproducibility (Winterton et al., 2021).

In this field, two recent reviews are strongly recommended. The First one focused on the potential application of IAO for treatment of mental diseases associated with altered socio-emotional competence, mainly in the mental health crisis associated to the Covid-19 pandemic (Grinevich and Neumann, 2021). In the second one, advances in previously debated methodological issues, such as reproducibility, statistical power, interpretation of non-significant results, dosage and sex differences are discussed, in order to suggest future directions for the translation of IAO into psychiatric applications (Quintana et al., 2021).

Keeping in mind these considerations, some findings regarding social-affective correlates of OXT using IAO will be mentioned. For instance, there is evidence that suggests the central system of oxytocin is altered in different mental disorders that are characterized as social disorders, personality disorders and early traumas. Regarding that, it has been found that the IAO in patients with autism spectrum disorders (ASD), schizophrenia and borderline personality disorder, improves performance in social cognition tasks that measure the ability to comprehend affective content, recognition of others’ emotions, social and facial perception, sarcasm comprehension and empathy (Heinrichs and Domes, 2008).

Another example is the study from Heinrichs et al. (2003) in which participants were randomly assigned a IAO treatment or a placebo, 50 min before being exposed to a psychological stress condition. Additionally, the design included receiving or not support from each participants’ best friend during the stage of stress preparation. The participants who received social support and IAO exhibited the lowest cortisol concentration during the exposure, while those who did not receive social support exhibited higher cortisol levels.

On the other hand, in a study that implemented functional magnetic resonance imaging (fMRI) in humans, Kirsch et al. (2005) observed the amygdala activation through a visual stimulus of fear induced in healthy males. Those who received IAO presented a lower emotional response than those who received placebos. The conclusion was reached, that OXT could have a modulatory role in the amygdala response to emotions of negative valence.

Kosfeld et al. (2005) showed that an only dose IAO causes a substantial increase in the trust of human beings, increasing the benefits of social interaction in a trust game. This study showed that the effect of OXT in trust was not due to a general increase in the disposition to adopt risk. In other words, OXT increases an individual's disposition to accept social risks in social interactions.

A study conducted by Domes et al. (2007) examined the effects of IAO in the ability to infer the affective state of an individual through facial signals. In this study, participants were given a set of photographs showing the eye region of different facial expressions and were asked to tell the state of the person. A single dose of 24 UI OXT administered intranasally improved performance during this test in comparison with the placebo. In this way, the induction of OXT could facilitate the performance of determined cognitive functions that might be associated with social approach (Heinrichs and Domes, 2008).

Additionally, it has been documented that IAO modulates the action of other hormones such as cortisol (Quirin et al., 2011; Weisman et al., 2013a, Weisman et al., 2013b) and testosterone (Weisman et al., 2014). Likewise, cortical activity has been affected by IAO, in both EEG and fMRI studies, especially regarding the connection between the amygdala and prefrontal cortex (Sripada et al., 2013; Dodhia et al., 2014; Ebner et al., 2016), and the activity in the caudate nucleus, cingulate and visual cortex (Li et al., 2017).

Finally, it has been found that the administration of IAO increases the expression of prosocial skills and behaviors such as parental care (Weisman et al., 2013a, Weisman et al., 2013b; Weisman et al., 2014; Gordon et al., 2017; Li et al., 2017), empathy (Bartz et al., 2010; Feeser et al., 2015; Strang et al., 2017), perception of facial expressions (Savaskan et al., 2008) and cooperation (Ten Velden et al., 2017). However, other authors also found that IAO was associated to negative social emotion like envy and “schadenfreude” (gloating), which suggested a possible role of OXT modulating social emotions and behaviors that could be more complex than classical association with prosociality and positive emotions (Shamay-Tsoory et al., 2009).

In sum, regarding the studies described, OXT modulates social perception, social cognition and social behavior, possibly promoting the approach between individuals and affiliation. These effects might be associated with its capacity to modulate functions linked with emotional processing and recognition. Additionally, functional imaging studies support the idea that anxiolytic effects related to the exogenous OXT administration are due, at least partially, to the deactivation of amygdala excitation. Therefore, reduction in emotional excitation during social encounters might foster social approach and contribute to the positive effects of OXT in trust and social cognition.

## 11 Conclusions

Different techniques have enabled the rigorous study of OXT since its discovery in 1909, and, even though more than 100 years have passed, its effect on behavior is still assessed. Despite findings in immunochemical techniques, some difficulties persist in making an optimal measurement of OXT, placing at risk the validity and scope of the experiments requiring this type of analysis.

Different samples and methods could yield a wide range of results, that vary from 1 to nearly 1000 pg/ml, and this makes their comparison and the establishment of clear trends and conclusions difficult. Distinct protocols of extraction and processing of fluid samples could also introduce differences in OXT measurements. Furthermore, many authors report their results in different measurement units (pg/ml, pM, pg/mg of salivary protein, pmol/l, uU/ml), which makes data comparison, extrapolation and discussion harder. Some studies do not even report the absolute values of OXT, but only percentages of variation or levels of statistical significance. On the other hand, given the different ranges of absolute values, the levels of statistical significance in comparative analyzes can change dramatically.

As for IAO techniques, which are widely used today, they can induce false increases in salivary OXT levels by a direct passage of the exogenous substance to the oropharynx. At other times, elevated peripheral oxytocin levels may be associated with the measurement of other substances chemically similar to the hormone, such as other neuropeptides.

In addition, the complexity of measuring OXT in a standardized way is not only related to the measurement methods used, but to many other physiological factors that can induce changes in its concentration and limit behavioral and psychological correlations. Among these, there are not only hormonal interaction phenomena that can affect OXT levels, but also possible interactions with other chemical signals, as shown by studies in which allergy sufferers have higher baseline levels of oxytocin in their blood (Glenk et al., 2020). All of these aspects make it quite difficult to compare different results of studies and to correlate the data they yield with behavioral or psychological parameters.

Despite the fact that one of the most used protocols in recent years for the measurement of saliva OXT is that of Carter et al. (2007), the same authors in their method validation article acknowledge that:

“However, the amounts of OXT present were low and the methods used here for concentrating samples are labor-intensive. A more sensitive assay is desirable, if salivary OXT is to become a readily available “biomarker” for use in studies of human behavior or physiology”.

Probably, changes in the treatment of salivary samples and monoclonal antibody use might be the key to obtain more reliable results. Some authors recently suggest that other methods like liquid chromatography-mass spectrometry (LC-MS) could yield better results to measure salivary OXT (Wang et al., 2019), but expenses and availability of this technology could strongly limit its wide research use. In any case, the field of research on the biological, psychological and social functions of OXT continues to expand, and for this reason it becomes imperative to reach better agreements on the techniques and measurement methods used.

## Declarations

### Author contribution statement

All authors listed have significantly contributed to the development and the writing of this article.

### Funding statement

This work was supported by Universidad Externado de Colombia.

### Data availability statement

No data was used for the research described in the article.

### Declaration of interests statement

The authors declare no conflict of interest.

### Additional information

No additional information is available for this paper.
